# Benefits of Brief Group Cognitive Behavioral Therapy in Reducing Diabetes-Related Distress and HbA1c in Uncontrolled Type 2 Diabetes Mellitus Patients in Thailand

**DOI:** 10.3390/ijerph17155564

**Published:** 2020-08-01

**Authors:** Kongprai Tunsuchart, Peerasak Lerttrakarnnon, Kriengkrai Srithanaviboonchai, Surinporn Likhitsathian, Sombat Skulphan

**Affiliations:** 1 Department of Community Medicine, Faculty of Medicine, Chiang Mai University, Chiang Mai 50200, Thailand; t.kongprai@gmail.com (K.T.); kriengkrai.s@cmu.ac.th (K.S.); 2 Department of Family Medicine, Faculty of Medicine, Chiang Mai University, Chiang Mai 50200, Thailand; 3 Research Institute for Health Sciences, Chiang Mai University, Chiang Mai 50200, Thailand; 4 Department of Psychiatry, Faculty of Medicine, Chiang Mai University, Chiang Mai 50200, Thailand; surinporn.l@cmu.ac.th; 5 Department of Psychiatric Nursing, Faculty of Nursing, Chiang Mai University, Chiang Mai 50200, Thailand; sombat.sk@cmu.ac.th

**Keywords:** cognitive behavior therapy, diabetes-related distress, type 2 diabetes mellitus

## Abstract

This study evaluated the short-term efficacy of brief group cognitive behavioral therapy (BG-CBT) in reducing diabetes-related distress (DRD), lowering hemoglobin A1c (HbA1c), improving food consumption behavior, increasing physical activity, and improving medication adherence behavior. A quasi-experimental pretest/post-test design with follow-up assessments was used with an experimental and a control group. Participants were patients with uncontrolled type 2 diabetes mellitus (T2DM) and moderate or high diabetes-related distress recruited from the Diabetes Mellitus Clinic of Hang Dong Hospital, Chiang Mai, Thailand. Fifty-six eligible participants were purposively selected and enrolled, then randomly assigned to either the BG-CBT group or the control group. The BG-CBT group received six brief weekly sessions of cognitive behavioral group therapy, while the control group received conventional care. Baseline data were collected at week 0 (pretest) and at week 6 (post-test), including food consumption behavior, physical activity, and adherence to medication regimes, as well as a blood examination to determine levels of HbA1c at the week 12 follow-up. DRD was assessed using the Diabetes Distress Scale (DDS-17) and analyzed using descriptive statistics, including pair t-test and independence t-test results. The BG-CBT had a significant effect on the amelioration of diabetes distress, improvement of food consumption behavior, and reduction of HbA1c levels, demonstrating the effectiveness of BG-CBT in maintaining diabetes control in people with T2DM-related distress.

## 1. Introduction

Globally, diabetes mellitus is one of the most common metabolic disorders with a prevalence estimated to be 9.3% (463 million) and expected to rise to 10.9% (700 million) by 2045. The prevalence of impaired glucose tolerance is estimated to be 7.5% (374 million) in 2019 and is projected to reach 8.6% (548 millions) by 2045, with type 2 diabetes mellitus (T2DM) accounting for approximately 90% of that total [1]. In Thailand, the prevalence of T2DM is currently 9.9% of the adult population [2], higher than the global prevalence.

Many adults with T2DM experience a psychosocial burden and mental health problems associated with the disease [3]. The daily demands of people living with T2DM increase their risk of diabetes-related distress (DRD), a condition of stressful feelings associated with the challenges of managing diabetes and concerns related to diabetes complications [4].

DRD is a syndrome comprised of multidimensional components, including worry, conflict, frustration, and discouragement that can accompany living with diabetes [5]. The prevalence of DRD is reported to be 18–45%, with an incidence of 38–48% over 18 months [6]. High levels of diabetes distress can significantly impact medication adherence behavior and is linked to higher hemoglobin A1c (HbA1C) levels, lower self-efficacy, and poorer dietary and physical activity behaviors [7,8,9,10]. Previous studies have also reported that long-term DRD is associated with anxiety [11] and depression [12,13,14,15]. DRD has been shown to impact type 2 diabetes patients negatively through poor adherence to medication regimens and through reduced self-care [16,17]. A distinction has been drawn between DRD and depression, with research suggesting that DRD is more widespread [17]. The literature further suggests that DRD has a greater impact upon and is more closely associated with diabetes self-management and diabetes-related behavioral and biomedical outcomes than depression [8]. DRD is not a mental pathology or a common psychiatric disorder, which, when diagnosed, can be treated with medication [18]. Since DRD is linked to specific stressors, and much of diabetes distress is an expected reaction to a serious and chronic health-related stressor, it is viewed as part of the spectrum of diabetes, not as a separate clinical condition indicating psychopathology [17]. A primary goal is to reduce DRD in order to make it possible for patients to control their blood-sugar levels appropriately. However, to date no such programs have been developed for the management of DRD.

Cognitive behavior therapy (CBT) is a form of psychotherapy that can help reduce potentially detrimental emotions, behavior, and physiology physiological responses [19]. It is widely used to treat psychiatric disorders, psychological problems, medical problems with a psychological component, comorbid psychiatric disorders, difficulties in adjustment to illness, poor adherence to treatment and other illness-related behavioral problems [20]. CBT has been applied in diabetic patients with depression—e.g., Safren et al. [21] and Aguilera et al. [22]—including diabetic patients with DRD. Amsberg et al. [23] and Cummings et al. [24] report experimental studies of diabetic patients who could not control their blood-sugar level and who also had DRD found significant improvement in HbA1c levels and reduction of diabetes-related distress among the CBT intervention group compared with the control group. Similarly, Seyed et al. [25] conducted a quasi-experimental study of diabetic patients who could not control their blood-sugar levels and who had DRD. Their results indicated that CBT had significant effects on the amelioration of diabetes distress and on levels of HbA1c.

Although there is evidence that CBT can improve behavioral outcomes and may also improve HbA1c in patients with T2DM and DRD, including a number of studies using CBT in DRD therapy in patients with T2DM who could not control their blood-sugar levels, no such studies have been conducted in Thailand. The objective of this study was to develop and evaluate a Brief Group Cognitive Behavior Therapy (BG-CBT) program specifically aimed at reducing DRD, improving food consumption behavior, increasing physical activity, improving medication adherence, and controlling blood-sugar levels among Thai T2DM patients.

## 2. Materials and Methods

### 2.1. Study Design

A quasi-experimental study design was used to test the efficacy of the BG-CBT intervention. The study compared a 6-session weekly brief group cognitive behavioral therapy with conventional care control (general counseling) using a pretest/post-test design with post treatment follow-up.

### 2.2. Participants

**Figure 1:**
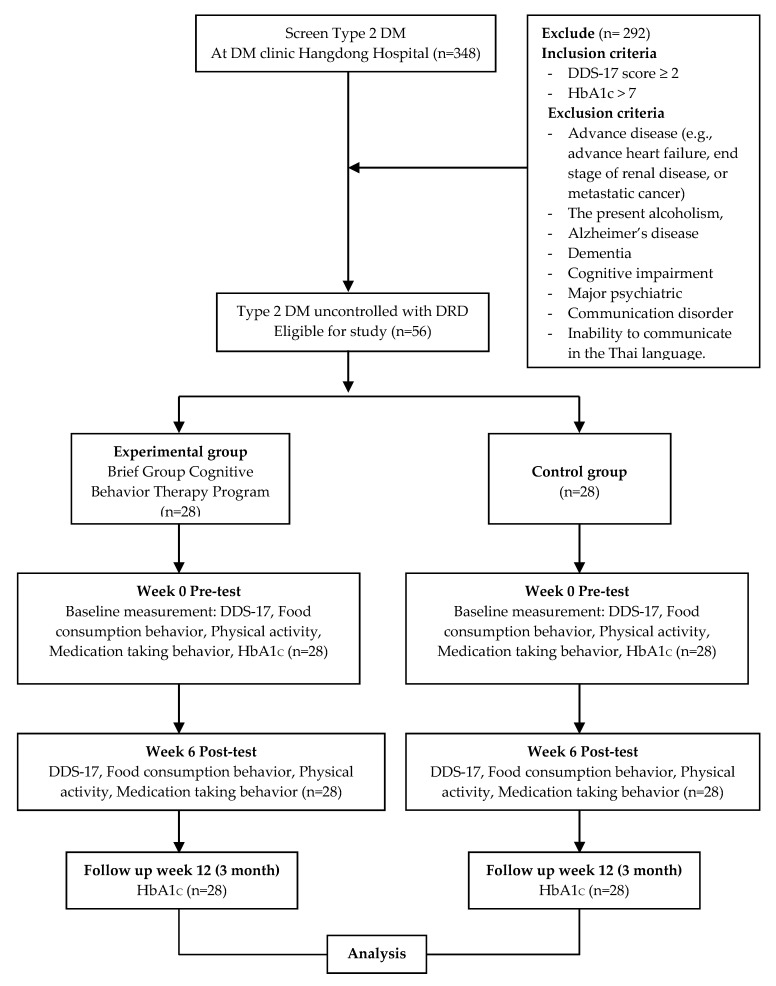
Flow chart of study procedure.

Participants were recruited from the DM clinic in Hang Dong Hospital, Chiang Mai Province, Thailand (Figure 1). Inclusion criteria were T2DM, age ≥ 18 years, HbA1c > 7, and DDS-17 score ≥ 2. Exclusion criteria were an established diagnosis of an advanced disease (e.g., advance heart failure, end stage renal disease, and metastatic cancer) or the presence of alcoholism, Alzheimer’s disease, dementia, cognitive impairment, a major psychiatric or communication disorder, and inability to communicate in the Thai language.

The study was comprised of 56 participants. The generally accepted standard for a quasi-experimental study is a minimum of 30 individuals with 20–25 participants for each independent variable [26]. Twenty-eight of the participants were randomly assigned to the experimental group, and the other 28 were assigned to the control group. The groups were matched for gender, age, DDS-17 score and HbA1c level.

### 2.3. Brief Group Cognitive Behavior Therapy (BG-CBT) Intervention Program

The 6-session BG-CBT program was developed by the investigators and was based on Beck’s (2011) work [19]. Each weekly session was 60–90 min long. The program was reviewed by a panel of three CBT experts: one psychiatrist and two psychiatric nurses. The objectives of the first session included building relationships and understanding the connections between stimuli, thoughts, emotions, behavior, and physiology. The second session focused on identifying stimuli that promote inappropriate behavior and methods to modify that behavior including finding ways to manage stimuli. The third session attempted to identify inappropriate behavior affecting emotions, thoughts, and physiology, as well as the influence of external stimuli, including searching for methods to change inappropriate behavior. The forth focused on negative automatic thoughts. In the fifth, negative automatic thoughts to be improved or modified were selected. In the sixth and final session, knowledge and understanding of what had been learned was summarized, including systems for monitoring achievement of the mutually established goals.

Prior to leading the BG-CBT sessions, the therapist participated in a three-day brief cognitive behavioral group therapy intervention training program and also received approximately six hours of guidance by instructors of the Faculty of Nursing, Chiang Mai University, who are experts in CBT. The guidance included observation and postsession feedback, as well as case conferences. In addition, members of the research study team randomly observed the interventionist therapists during one or more of the sessions.

### 2.4. Outcomes and Measurement

#### 2.4.1. Primary Outcomes

Diabetes-related distress (DRD) was evaluated using the Diabetes Distress Scale (DDS-17) developed by Polonsky et al. [27], which had been translated into the Thai language by Kattika [5]. The questionnaire, which has an alpha coefficient of 0.95, is composed of 17 items and measures four critical dimensions of distress: emotional burden, regimen distress, interpersonal distress, and physician distress. Each item dimension is rated on a 6-point scale from 1 (no problem) to 6 (serious problems). Each dimension is interpreted as the mean score of each subcomponent: ˂2 = little or no distress, 2–2.9 = moderate distress, and ≥3 a high level of distress.

#### 2.4.2. Secondary Outcome

Food Consumption Behavior, the survey of food consumption behavior questionnaire developed by the Bureau of Nutrition, Department of Health, Ministry of Public Health, Thailand was used to assess the food consumption behavior of the participants. The Cornbrash’s alpha coefficient of the questionnaire is 0.75. The participation in each of the 14 survey items is divided into 3 frequency levels: daily (5–7 days per week—5 points), occasional (1–4 days per week—3 points), and never (0 points). The maximum total score is 100 points (very good habits), 80–99 (good habits), 60–79 (moderately good habits), <60 points (food consumption habits need improvement).

Physical activity was assessed using the Global Physical Activity Questionnaire (GPAQ) Version 2, developed by Armstrong et al. [28] and translated into the Thai language by Vanida [29] The questionnaire, with an alpha coefficient of 0.77, is composed of 16 items divided into 4 groups: group 1—occupational activities (6 items); group 2—activities related to traveling from one place to another (3 items); group 3—activities to do during free time for relaxation, resting, or recreation (6 items); and group 4—sitting and lying down behavior measured in metabolic equivalents (METs) (1 item).

Medication taking behavior evaluation of medication adherence behavior was measured using the Thai language Medication Taking Behavior Scale (MTB-Thai) [30]. The alpha coefficient of the questionnaire was 0.76. The questionnaire covered 6 areas: (1) forgetting to take medicines, (2) not taking medicines at the times prescribed, (3) stopping medicines because of adverse drug reactions, (4) stopping medicines because of getting better, (5) stopping medicines for other reasons, and (6) adjusting dosage regimens. Each item was rated on a scale of 1 to 4, then the ratings were combined. Scoring was as follows: high drug adherence = 24, medium drug adherence = 22–23, and low drug adherence ≤ 21.

Blood hemoglobin A1C (HbA1c) level was measured by ion-exchange High-Performance Liquid Chromatography (HPLC) at the Hang Dong Hospital Laboratory, Chiang Mai Province, Thailand.

### 2.5. Ethical Considerations

All participants provided written informed consent prior to participation in the study. The study protocol was approved by the Research Ethics Committee of the Faculty of Medicine, Chiang Mai University, Thailand (no. 357/2016).

### 2.6. Data Analysis

Demographic variables were analyzed using descriptive statistics; differences between groups were evaluated using the independent t-test. Differences within groups were analyzed using the paired t-test. Between-group comparisons were made using the independent t-test. *p*-Values < 0.05 were regarded as statistically significant.

## 3. Results

The 56 participants in this study (experimental group = 28, control group = 28) had an average age of 56.04 years (±8.33). Most (58.9%) were female and most had comorbidities (73.2%) or DM complications (46.4%). The average duration of their T2DM was 8.87 ± 6.79 years. The mean HbA1c level, diabetes-related distress, food consumption behaviors, physical activity, and medication taking behaviors were 9.56 ± 1.86, 2.46 (± 0.44), 49.00 (± 8.04), 76.73 (± 76.85), and 21.75 (± 1.98), respectively. There were no statistically significant differences between the two groups in age, gender, DM complications, comorbidities, duration of diabetes, DDS-17 score, HbA1c level, physical activity level, or medication taking behavior score. There was, however, a significant difference in food consumption behavior at baseline (Table 1).

Paired t-test analysis of pretest and post-test results in the experimental group and control group found a statistically significant difference in DDS-17 scores (*p* = 0.00). T2DM patients with DRD in the experimental group had improved food consumption behavior scores (*p* = 0.00) and HbA1c levels (*p* = 0.00) after receiving BG-CBT. In the control group, there was no statistically significant change in DDS-17 scores, consumption behavior scores, physical activity scores, medication behavior scores or HbA1c levels between pretest and post-test. This may be because there were no significant differences in changes in physical activity or medication taking behavior between the groups (Table 2).

Table 3 shows the changes in the primary and secondary outcome measures between the experimental group and the control group from baseline to 6 weeks (3 months for HbA1c) after receiving BG-CBT. The primary outcome—reduction in DRD as measured by DDS-17 score in the experimental group compared to the control group—reached statistical significance. For the secondary outcomes—consumption behavior and HbA1c levels—there were significant differences between the groups (*p* = 0.003 and *p* = 0.009, respectively). There was no difference in the mean difference for physical activity or medication taking behavior score between the experimental group and the control group.

## 4. Discussion

This first experimental study in Thailand found that BG-CBT could significantly reduce DDS scores (from 2.47 ± 0.41 to 2.08 ± 0.49) and HbA1c levels (from 9.44 ± 1.63 to 7.34 ± 1.10), as well as improve food consumption behavior scores (from 45.14 ± 7.37 to 50.21 ± 7.03) at the six-week follow-up (3 months for HbA1c levels) compared to the control group. There was improvement in the physical activity and medication taking behavior scores (from 69.90 ± 57.43 to 76.89 ± 61.39 and from 21.50 ± 2.03 to 21.89 ± 1.97, respectively), although the differences were not statistically significant. These results are consistent with a study by Seyed et al. [25], which reported on cognitive behavioral therapy (CBT) for diabetes patients and found that the pre-test to post-test changes in blood-sugar levels were significantly different in the two groups (experimental and control groups), and the level of diabetes distress in the experimental group before and after showed a difference, from 2.96 before therapy to 2.18 after therapy, which means that the level of diabetes distress decreased after therapy, which can significantly reduce blood-sugar levels and the degree of diabetes distress. There have been few studies explicitly comparing food consumption behavior before and after CBT in patients with type 2 diabetes, but research has found that using CBT results in improved dietary quality [31]. Most importantly, CBT has been shown to clarify misunderstandings that could lead to emotional distress and problem behavior [20].

This study shows that BG-CBT can reduce DRD, HbA1c levels, and improve food consumption behavior significantly compared to mean change of experimental group and control group conventional care alone. These results are consistent with a previous systematic review of randomized controlled trials of psychological interventions to improve glycemic control in people with type 2 diabetes. Eight of the twelve trials studies in that meta-analysis were involved in CBT and all eight reported improvement in long-term glycemic control and reduction of psychological distress in the CBT groups [32]. Similarly, several previous studies, e.g., Cummings et al. [24], evaluated the effect of cognitive behavioral therapy (CBT) plus lifestyle counseling in primary care on HbA1c levels in rural adult patients with T2DM with comorbid depressive and/or medication adherence behavior-related distress symptoms. The Cummings study found that patients in the intervention group showed significant improvement in HbA1c levels and greater improvement in DRD compared to the group receiving standard care. A study by Seyed et al. [25] on the effectiveness of combined group Cognitive Behavioral Therapy (CBT) in improving diabetes distress and glycemic control among 60 adults with type 2 diabetes found that reduced HbA1c levels were correlated with a reduction in DRD. That study found that the CBT had a significant effect on the amelioration of diabetes distress. Studies by Amsberg et al. [23] and Van et al. [33] of the effects of CBT on HbA1c and DRD in type 1 diabetic patients found significant differences in HbA1c and DRD between the experimental (CBT) group and the control group. In contrast, a randomized controlled trial by Welschen [34] on the effects of a cognitive behavioral treatment when added to managed care on consumption behavior in patients with type 2 diabetes found no statistically significant differences in eating behavior at three post-treatment time points. Similarly, a study of CBT by Yomogida et al. [35] found no statistically significant change in the diets of type 2 diabetes patients.

The present study found no significant differences in medication taking behavior or physical activity between the experimental group and control group, although medication taking behavior did improve in the experimental group. A study by Cummings et al. [24] found that patients receiving CBT intervention evidenced significantly greater improvements in medication adherence than those receiving the usual care. There may be other factors, however, that affect medication taking behavior. For example, in this study data were not collected on the patients’ medication knowledge [36], education level, income, perceived treatment inefficacy, treatment complexity or on hypoglycemia [37]. This study did find increased physical activity in both the experimental group and the control group, but the difference between the groups was not significant. That finding is consistent with a previous study that also reported no statistically significant differences in physical activity between the CBT treatment group and the control group [35]. In contrast, a systematic review of research reports on cognitive therapies concluded that the therapies probably lead to a moderate increase in physical activity compared to no intervention or to usual care, suggesting that cognitive therapy may have a slight impact on physical activity [38].

An advantage of brief CBT is that, because it is a group intervention, i.e., it treats several people at one time, it is more cost-effective and, thus, potentially more accessible to a greater number of individuals in need of assistance. Additionally, patients enjoy rapid treatment gains, which may improve the credibility of the treatment and, as a consequence, increase their motivation for further change [39]. Brief CBT also provides an opportunity for patients to learn from the experiences and homework tasks of other group members—so-called vicarious learning. Additionally, groups can be less stigmatizing for some and any stigma that does arise can be reduced by the ‘normalization effect’ of meeting others with the same problem. CBT groups provide a ready ‘audience’, which can provide exposure and the opportunity for behavioral experiments. The views of other group members often carry more weight or are viewed as more ‘neutral’ than the views of the therapists, although the therapists’ views are useful for cognitive challenging. CBT groups can also be beneficial for people who struggle with a one-to-one professional relationship, e.g., individuals who are likely to form a regressive and very dependent relationship with the therapist. Other patients who feel uncomfortable with a one-to-one professional relationship in therapy may prefer group work [40].

A limitation of this study was that it was not randomized, so results might have been affected by unknown confounding variables. There may also have been contamination of the sample (sampling bias), because the study involved a group of patients treated at the same diabetes clinic. A randomized control trial is warranted to confirm the effectiveness of a brief CBT program. Also, the results were monitored for only a short period of time, so the long term sustainability of the effects were not determined. The study has not collected data on the types of drugs that patients used to treat diabetes: some drugs (e.g., metformin) may affect cognitive deficit [41,42]. Therefore, future studies should collect data on medications used to treat diabetes. The effect of this intervention for older adults with T2DM has not been shown in this study and should be studied further.

## 5. Conclusions

BG-CBT is effective in reducing DRD, lowering HbA1c levels and improving food consumption behavior in Thai populations compared to standard treatment. The potential importance of the findings suggests that a larger-scale study be conducted, incorporating further methodological refinements, e.g., expanding the locations of sample collection, including a larger sample and conducting an extended follow-up assessment.

## Figures and Tables

**Table 1:** Baseline characteristics of participants.

Parameter	Experimental Group (*n* = 28)	Control Group (*n* = 28)	Total (*n* = 56)	*p*-Value
Age (years) mean ± SD	58.18 ± 8.83	53.89 ± 7.34	56.04 ± 8.33	0.093
Gender (% female)	57.10%	60.70%	58.90%	0.813
DM complications (% present)	32.10%	60.70%	46.40%	0.326
Comorbidities (% present)	85.70%	60.70%	73.20%	0.713
Duration of diabetes (years) mean ± SD	10.34 ± 7.77	7.39 ± 5.40	8.87 ± 6.79	0.077
HbA1c% mean ± SD	9.44 ± 1.63	9.68 ± 2.09	9.56 ± 1.86	0.575
DDS-17 scores mean ± SD	2.46 ± 0.41	2.46 ± 0.47	2.46 ± 0.44	0.915
Physical activity mean ± SD	69.90 ± 57.43	83.56 ± 92.92	76.73 ± 76.85	0.511
Food consumption behavior score mean ± SD	45.14 ± 7.37	52.86 ± 6.81	49.00 ± 8.04	0.000
medication taking behavior score mean ± SD	21.50 ± 2.03	22.00 ± 1.94	21.75 ± 1.98	0.350

**Table 2:** Comparison of pretest and post-test within group means (paired t-test).

Parameter	Experimental Group (*n* = 28), Mean (± SD)		*p*-Value	Control Group (*n* = 28), Mean (± SD)		*p*-Value
	Baseline	Post Intervention		Baseline	Post Intervention	
DDS score	2.47 ± 0.41	2.08 ± 0.49	<0.001	2.46 ± 0.47	2.48 ± 0.80	0.817
Food consumption behaviors score	45.14 ± 7.37	50.21 ± 7.03	<0.001	52.86 ± 6.81	52.50 ± 5.07	0.777
Physical activity	69.90 ± 57.43	76.89 ± 61.39	0.176	83.56 ± 92.92	92.43 ± 109.88	0.372
Medication taking behaviors score	21.50 ± 2.03	21.89 ± 1.97	0.110	22.00 ± 1.94	21.46 ± 2.91	0.216
HbA1c%	9.44 ± 1.63	7.34 ± 1.10	<0.001	9.68 ± 2.09	8.97 ± 2.38	0.101

**Table 3:** Mean changes in outcomes from baseline to six-week follow-up (3 months for HbA1c levels).

Parameter	Experimental Group (*n* = 28) Mean (± SD)			Control Group (*n* = 28) Mean (± SD)			*p*-Value Change
	Baseline	Follow-up	Mean Change	Baseline	Follow-up	Mean Change	
DDS score	2.47 ± 0.41	2.08 ± 0.49	−0.39 ± 0.47	2.46 ± 0.47	2.48 ± 0.80	+0.02 ± 0.62	0.006
Food consumption behaviors score	45.14 ± 7.37	50.21 ± 7.03	+5.07 ± 6.57	52.86 ± 6.81	52.50 ± 5.07	−0.36 ± 6.60	0.003
Physical Activity	69.90 ± 57.43	76.89 ± 61.39	+6.99 ± 26.64	83.56 ± 92.92	92.43 ± 109.88	+8.87 ± 51.67	0.865
Medication taking behaviors score	21.50 ± 2.03	21.89 ± 1.97	+0.39 ± 1.26	22.00 ± 1.94	21.46 ± 2.91	−0.54 ± 2.24	0.062
HbA1c%	9.44 ± 1.63	7.34 ± 1.10	−2.10 ± 1.53	9.68 ± 2.09	8.97 ± 2.38	−0.71 ± 2.21	0.009
